# ROCK Inhibition Activates MCF-7 Cells

**DOI:** 10.1371/journal.pone.0088489

**Published:** 2014-02-11

**Authors:** Seungwon Yang, Hyun-Man Kim

**Affiliations:** Laboratory for the Study of Molecular Biointerfaces, Department of Oral Histology and Developmental Biology, Program of Cell and Developmental Biology, School of Dentistry and Dental Research Institute, Seoul National University, Seoul, Republic of Korea; Karolinska Institutet, Sweden

## Abstract

Dormant carcinoma cancer cells showing epithelial characteristics can be activated to dissipate into the surrounding tissue or organs through epithelial-mesenchymal transition (EMT). However, the molecular details underlying the activation of dormant cancer cells have been less explored. In this study, we examined the molecular pathway to activate dormant breast cancer cells. Rho-associated kinase (ROCK) inhibition disrupted cell junction, promoted cell proliferation and migration / invasion in both two-dimensional and three-dimensional substrates. The disintegration of cell junction upon ROCK inhibition, coupled with the loss of E-cadherin and b-catenin from the cell membrane, was associated with the activation of Rac1 upon ROCK inhibition. Migration / invasion also increased upon ROCK inhibition. However, the activation of MCF-7 cells upon ROCK inhibition was not associated with the up-regulation of typical EMT markers, such as snail and slug. Based on these results, we suggest the potential risk for dormant cancer cells to dissipate through non-typical EMT when ROCK activity is down-regulated.

Epithelial-originated cancer cells, such as breast cancer cells, transition between phases of epithelial and mesenchymal cells during pathological progression [1], [2]. Initially, cancer cells transit from epithelial cells to mesenchymal cells through epithelial-mesenchymal transition (EMT), which is a critical step required for metastasis. After leaving the epithelium as mesenchymal cells through EMT, cancer cells reversibly transform into epithelial cells through mesenchymal-epithelial transition (MET). Cancer cells transformed from the mesenchymal phase to epithelial phase, which re-establishes the epithelial phenotype, such as cell-to-cell attachment through E-cadherin, might, in turn, enter the dormant stage at remote sites from the original site [3], [4]. Dormant cancer cells arrest in cell cycle or exist for long time in a balance between proliferation and apoptosis. However, dormant cancer cells might be activated to dissipate further or metastasize through EMT. Thus, the EMT of dormant epithelial cancer cells might disseminate cancer cells in a similar manner as the EMT of the original epithelial cells. Breast cancer is a well-known cancer, which progressing through the dormant phase [5], [6]. Thus, understanding the molecular mechanisms of EMT in dormant breast cancer cells might provide information concerning the pathogenesis of breast cancer metastasis.

Rho-associated kinase (ROCK), a downstream of small RhoA GTPase (RhoA), regulates the cytoskeleton through the regulation of actin-myosin interactions [7], [8]. Recently, however, other roles for ROCK are emerging. ROCK is associated with various cellular activities such as cell proliferation, migration, and survival. ROCK activity is highly activated to suppress cell cycle progression and cell migration when adhesion signaling is weak [9]–[12]. Furthermore, ROCK inhibition promotes cell proliferation through the down-regulation of phosphatase and tension homolog (PTEN) and the up-regulation of Akt phosphorylation [10], [11] or accelerates cell migration through the activation of Rac1 [12]. In the present study, we propose that the previous findings might explain the dormancy of tumor cells, manifested in cells that are not properly attached to the extracellular matrix (ECM) [13]. The ECM is recognized as a gatekeeper for the metastatic growth of dormant cancer cells. The metastatic growth of dormant cancer cells was inhibited when integrin receptors were blocked [13]. Thus, we hypothesize that ROCK activity, which reflects the status of adhesion signaling [9]–[12], might be closely associated with the activation of dormant cancer cells. In the present study, we demonstrated that the inhibition of ROCK activates dormant MCF-7 breast cancer cells of which ROCK activity is dependent on the adhesion strength. Furthermore, the potential undesirable effect of ROCK inhibition on the activation of dormant cancer cells is of interest, as ROCK inhibition has recently been considered for the control of cardiovascular diseases ascribed for the prevention of contracting cells [14]–[17].

## Materials and Methods

### Cell culture and reagents

The MCF-7 human breast cancer cell line was obtained from the American Type Culture Collection (ATCC, Manassas, VA). MCF-7 Cells were cultured on culture plates or in Matrigel (BD Bioscience, San Diego, CA) in the recommended medium supplemented with 10% fetal bovine serum (FBS, GIBCO BRL, Carlsbad, NY), 10 mg/ml insulin, 100 U/ml penicillin, and 100 mg/ml streptomycin (GIBCO BRL, Carlsbad, NY) at 37°C in 95% air and 5% CO_2_. The 3D cell culture was performed using the “3D on-top” method [18]. Briefly, prechilled cell culture dishes with 4-well chambers (Nunc, Penfield, NY) were coated with the growth factor-reduced Matrigel, thawed overnight at 4°C. MCF-7 cells were seeded as a single cell layer on Matrigel. Subsequently, the seeded cells were overlaid with culture medium containing 10% Matrigel to facilitate the 3D environment. MDA-MB-231 cells obtained from the American Type Culture Collection (ATCC, Manassas, VA) were cultured on culture plates in the recommended medium supplemented with 10% fetal bovine serum (FBS, GIBCO BRL, Carlsbad, NY), 100 U/ml penicillin, and 100 mg/ml streptomycin (GIBCO BRL, Carlsbad, NY) at 37°C in 95% air and 5% CO2.

The following specific pharmacological reagents were used to inhibit cell signaling: LY-294002 (Sigma-Aldrich, St. Louis, MO) for phosphatidylinositol 3-kinase (PI3-K) inhibition, cell-permeable C3 transferase (C3) (Cytoskeleton, Denver, CO) for RhoA inactivation, potassium bisperoxo (1,10-phenanthroline) oxovanadate (bpV(Phen)) (Calbiochem, La Jolla, CA) for phosphatase and tension homolog (PTEN) inhibition, Y-27632 (Tocris Cookson, Avonmouth, UK) for ROCK inhibition, and Rac1 inhibitor (Calbiochem, La Jolla, CA) for Rac1 inhibition. F-actins were stained with rhodamine-labeled phalloidin (Invitrogen, Carlsbad, CA). The nuclei were stained with DAPI (Sigma-Aldrich, St. Louis, MO). Fibronectin (Sigma-Aldrich, St. Louis, MO) was used to pre-coat the hydrophobic culture dishes before the culture of MCF-7 cells.

### Confocal laser microscopic observation

The cells were fixed with 4% paraformaldehyde/PBS for 30 min and permeabilized in 0.2% Triton X-100/PBS for 20 min. Subsequently, immunofluoresence staining was performed blocking the cells with 2% bovine serum albumin in PBS to reduce non-specific staining. Secondary staining was performed using anti-Rabbit Alex Fluor®488 (Cell Signaling Technology, Beverly, MA) or anti-rabbit Cy3 (Jackson ImmunoResearch Laboratories, West Grove, PA). The stained cells were observed under a LSM 700 confocal microscope (Carl Zeiss, Jena, Germany).

### Rac1 activity assay

The level of GTP-loaded Rac1 was determined using the G-LISA Rac1 activation assay kit (Cytoskeleton, Denver, CO) according to the manufacturer's instructions. Equal amounts of proteins from each experimental group were used for G-LISA Rac1 activation assay to obtain total Rac1 activity per cell.

### Immunoblotting

The cells were lysed with a lysis buffer (Cell Signaling Technology, Beverly, MA) containing 20 mM Tris-HCl (pH 7.5), 150 mM NaCl, 1 mM Na_2_EDTA, 1 mM EGTA, 1% Triton, 2.5 mM sodium pyrophosphate, 1 mM b-glycerophosphate, 1 mM Na_3_VO_4_, 1 µg/ml leupeptin, 1 mM PMSF, and a complete protease inhibitor cocktail tablet (Santa Cruz Biotechnology, Santa Cruz, CA). After high-speed centrifugation of the cell lysates, the protein concentration of the supernatants was determined using the Bradford assay (Bio-Rad, Hercules, CA). The proteins were run on sodium dodecyl sulfate polyacrylamide (SDS–PAGE) gels and transferred to polyvinylidene fluoride membranes. The membranes were blocked with 5% skim milk in TBS-T buffer (100 mM Tris-HCl pH 7.5, 150 mM NaCl, and 0.1% Tween-20) and subsequently incubated with primary antibodies overnight at 4°C, followed by incubation with secondary antibodies in 5% skim milk in TBS-T for 1 h at room temperature. The blots were developed using a horseradish peroxidase (HRP) chemiluminescent substrate reagent kit (Invitrogen, Carlsbad, CA). The following antibodies were used: anti-phospho-AKT (Ser 473) (Cell Signaling Technology, Beverly, MA), anti-glyceraldehyde 3-phosphate dehydrogenase (GAPDH) (Cell Signaling Technology, Beverly, MA), anti-PTEN (Santa Cruz Biotechnology, Santa Cruz, CA), anti-E-cadherin (Cell Signaling Technology, Beverly, MA), anti-b-catenin (Cell Signaling Technology, Beverly, MA), anti-EGFP (Biovision, Milpitas, CA), anti-Rac1 (Millipore, Billerica, MA), anti-flotillin-1 (Cell Signaling Technology, Beverly, MA), anti-snail (Cell Signaling Technology, Beverly, MA), anti-slug (Cell Signaling Technology, Beverly, MA), anti-b-actin (Cell Signaling Technology, Beverly, MA), anti-vimentin (Cell Signaling Technology, Beverly, MA), anti-N-cadherin (Cell Signaling Technology, Beverly, MA), and anti-occludin (Santa Cruz Biotechnology, Santa Cruz, CA).

### Transient and siRNA transfections

MCF-7 cells were cultured for 18 hrs before transient transfection with each plasmid using GenJet™ Reagent II for MCF-7 (Signagen, Ijamsville, MD) according to the manufacturer's instructions. The plasmid expression was confirmed through changes in cell shape, and immunoblot or fluorescence analyses. The following plasmids (Addgene, Cambridge, MA) were used: pCDNA3-EGFP (Addgene ID 13031), pCDNA3-EGFP-RhoA-Q63L (constitutively active RhoA, Addgene ID 12968), pCDNA3-EGFP-RhoA-T19N (dominant negative, Addgene ID 12967), pCDNA3-EGFP-Rac1-Q61L (constitutively active Rac1, Addgene ID 12981), pCDNA3-EGFP-Rac1-T17N (dominant negative, Addgene ID 12982), and pCDNA3-GFP-PTEN (wild type, Addgene ID 10759). For the RNA interference (RNAi) experiments, MCF-7 cells were seeded for 18 hrs and transfected with siRNA (Santa Cruz Biotechnology, Santa Cruz, CA) using a siRNA transfection reagent (Santa Cruz Biotechnology, Santa Cruz, CA) according to manufacturer's instructions. The siRNA for targeting ROCK-1, ROCK-2, and PTEN were obtained from Santa Cruz Biotechnology (Santa Cruz Biotechnology, Santa Cruz, CA).

### Subcellular fractionation

The subcellular fractionation of nuclear proteins and membrane proteins was performed using the Qproteome Cell Compartment Kit (Qiagen, Hilden, Germany) according to the manufacturer's instructions.

### Migration and invasion assay

To assay the migration and invasion of cells, 5×10^4^ cells were suspended in 150 ml of serum-free medium and seeded onto 8-mm Pore Transwell Inserts (Corning, Corning, NY) for the migration assay or 8-mm Pore Transwell Inserts coated with Matrigel for the invasion assay. The lower chambers were filled with 800 ml of complete medium. To terminate the migration of the cells, the cells on the Transwell Inserts were fixed with 4% paraformaldehyde/PBS for 30 min. Subsequently, the fixed cells were stained with hematoxylin solution (Sigma-Aldrich, St. Louis, MO) for 1 hr. After wiping off the cells on the upper side of the filter on the Transwell Inserts using cotton swabs, microphotograms of the cells migrated on the lower side of the filter were taken using light microscopy. Cells migrated or invaded the lower side of the filter were manually counted from the microphotograms. The mean cell number was obtained from five randomly selected squares of same area per the transwell insert and were compared statistically.

### Statistical analysis

The experimental data represents the mean ± standard deviation (SD) of at least three samples. Statistical analyses were performed for the comparison of the two groups using the paired t-test when the data were in normal distribution or signed rank sum test when the data were not in normal distribution. For the comparison of more than two groups, one-way analysis of variance was used when the data were in normal distribution, and the Kruskal-Wallis test was used when the data were not in normal distribution. A *P*-value <0.05 was considered statistically significant.

## Results

### MCF-7 cells is activated upon ROCK inhibition

**Figure 1:**
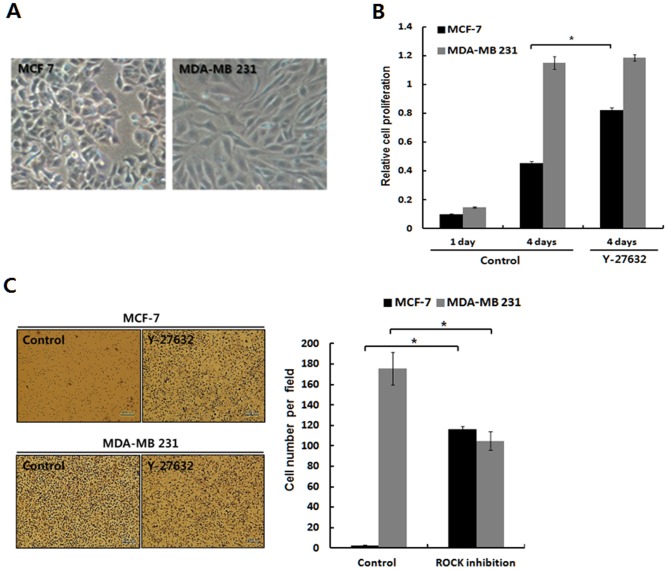
Cell activation upon ROCK inhibition is evident in low-active MCF-7 cells, not in highly active MDA-MB-231 cells. **A.** MCF-7 Cells growing in an epithelial fashion were cultured for three days, whereas MDA-MB-231 cells growing in a mesenchymal fashion were cultured for three days. **B.** MCF-7 cells and MDA-MB-231 cells were cultured on tissue culture plates for four days with or without Y-27632 (20 µM). Relative cell proliferation rates were determined by the cell number assayed using CCK-8 kit. The data are expressed as the mean ± SD. n = 3 culture dishes. The p-value was less than 0.05 for comparisons between control (Control) and treatment groups (Y-27632) in MCF-7 cells. *, *p*<0.05. **C**. The cells seeded on the Transwell chambers for the migration assay were incubated for 24 hrs in the absence or presence of Y-27632 (20 µM). The cells migrated into the lower chamber were manually counted after staining cells with hematoxylin. The data are expressed as the mean ± SD. n = 3 culture dishes. The p-value was less than 0.05 for comparisons between control (Control) and treatment groups (Y-27632). *, *p*<0.05.

First, the activities of the dormant epithelial MCF-7 cells and more proliferative MDA-MB-231 cells were monitored, as the responsiveness of the cells to the ROCK inhibition is dependent upon their activity [9]–[12]. The low active cells are up-regulated to the ROCK inhibition, whereas the highly active cells are not as responsive and are even down-regulated by the ROCK inhibition [9]–[12]. The dormant epithelial MCF-7 cells proliferate and migrate more slowly compared to the mesenchymal MDA-MB-231 cells [Fig 1]. Thus, the MCF-7 cells was supposed to respond more positively to the ROCK inhibition, while the MDA-MB-231 cells did not. In the assays of proliferation and migration, which are consistent with our conjecture that cellular responsiveness to ROCK inhibition is dependent on cellular activity, the activating effect of ROCK inhibition was effective for the low active epithelial MCF-7 cells but not for the metastatic MDA-MB-231 cells [Fig 1]. These results indicate that responsiveness to ROCK inhibition may be closely associated with the cellular activity in cancer cells as shown in normal cells.

### ROCK inhibition dissipates MCF-7 cells

**Figure 2:**
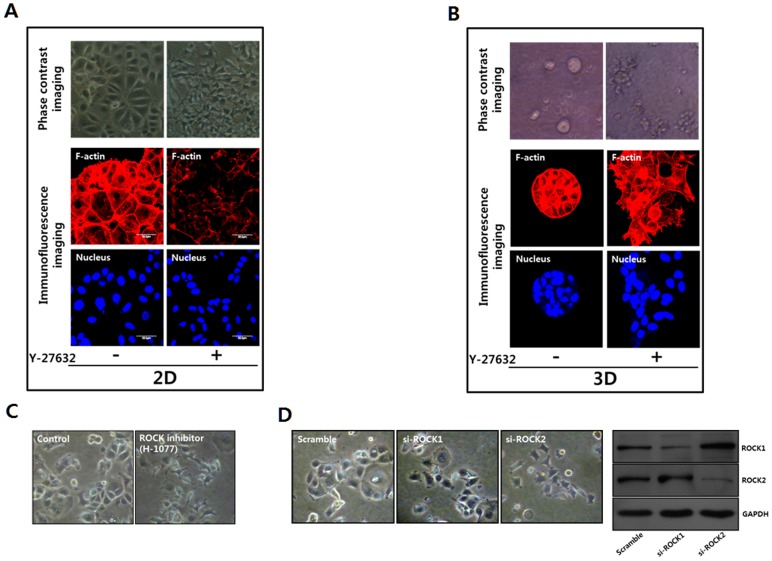
ROCK inhibition induces the scattering of MCF-7 cells in both 2D and 3D cultures. **A.** MCF-7 cells were seeded on tissue culture plates for culturing on two-dimensional surfaces (2D). Subsequently, the cells were cultured for 24 hrs in the absence or presence of Y-27632 (20 µM) to inhibit ROCK activity. The cells were plated in the absence of Y-27632 for 12 hours before treating cells with Y-27632 to inhibit ROCK activity. **B.** MCF-7 cells were cultured on a three-dimensional Matrigel matrix (3D) for 3 days. Subsequently, the cells were cultured for 3 or 4 days in the absence or presence of Y-27632 (20 µM) to inhibit ROCK activity (ROCK inhibition). The cells were observed through phase contrast microscopy or confocal laser microscopy after staining the fixed cells with rhodamine-phalloidin or DAPI to examine actin filaments (red) or nuclei (blue), respectively. **C.** The cells were cultured for 24 hrs in the absence or presence of H-1700 (30 µM) to inhibit ROCK activity and observed using phase contrast microscopy. **D.** MCF-7 cell morphology after transfection with control si-RNA (scramble), si-RNA targeting ROCK1 (si-ROCK1) or ROCK2 (si-ROCK2) was observed using phase contrast microscopy. The cells were incubated for 24 hrs after transfecting cells with control si-RNA (scramble), si-RNA targeting ROCK1 (si-ROCK1) or ROCK2 (si–ROCK2). Expression levels of ROCK1 and ROCK2 were measured using immunoblotting.

MCF-7 cells proliferated to form monolayer cell sheets in 2D culture and discrete spherules of cells in 3D Matrigel culture [Fig. 2]. The cell-to-cell junction was tight, as shown in the typical epithelium in both 2D and 3D cultures. MCF-7 cells lost epithelial growth patterns and were dissipated as single cells after ROCK inhibition using the specific pharmacological inhibitor, Y-27632, in both 2D and 3D cultures [Fig. 2]. The loss of epithelial characteristics upon ROCK inhibition was evident in the established pre-formed epithelial sheets or spherules and in cells treated with ROCK inhibitor from the beginning of cell culture. Specific pharmacological inhibitors of ROCK activity, such as Y-27632 [Fig. 2A and B] and H-1077 [Fig. 2C] or si-RNAs targeting ROCK1 or si-ROCK2 [Fig. 2D], induced similar results.

Because cytoskeletal alteration is suggested in the activation of dormant cancer cells [19], changes in the actin filaments were examined after ROCK inhibition [Fig. 2A and B]. The actin filament bundles developed underneath the cell membrane disappeared after ROCK inhibition, concomitant with the separation of cell junction in MCF-7 cells. However, upon ROCK inhibition, no development of stress fibers was observed, although it has been suggested that this development is required for the activation of dormant cancer cells [13], [19].

### ROCK inhibition in MCF-7 cells dislocates E-cadherin and b-catenin from the cell membrane

**Figure 3:**
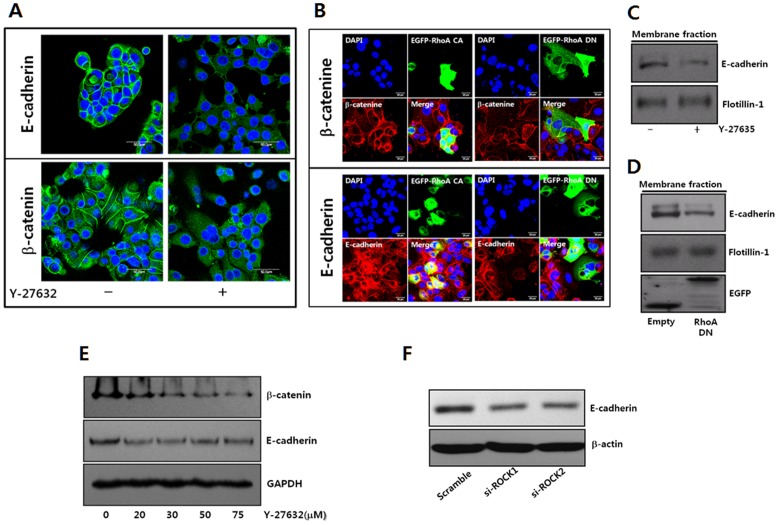
RhoA and ROCK activities regulate E-cadherin and b-catenin in MCF-7 cells. **A.** The localized patterns of E-cadherin and b-catenin were examined using specific antibodies through confocal laser microscopy. MCF-7 cells were cultured for 24 hrs in the absence or presence of Y-27632 (20 µM) to inhibit ROCK activity. **B.** The activity of RhoA, a major upstream regulating molecule of ROCK, was regulated to examine the role of RhoA-ROCK in regulating the membrane localization of E-cadherin and b-catenin after transfecting MCF-7 cells with constitutively active RhoA (EGFP-RhoA-Q63L, EGFP-RhoA CA) or dominant negative RhoA (EGFP-RhoA-T19N, EGFP-RhoA DN). E-cadherin and b-catenin were analyzed after staining the fixed cells with specific antibodies under confocal laser microscopy. The cells were also stained with DAPI to localize the nuclei. **C.** Membrane fraction level of E-cadherin was analyzed through immunoblotting using specific antibodies after treating MCF-7 cells with Y-27632 (20 µM) to inhibit ROCK activity for 24 hrs. An antibody to flotillin-1 was used to confirm the equal load of the membrane fraction. **D.** The level of E-cadherin in the membrane fraction was examined through immunoblotting using specific antibodies after transfecting MCF-7 cells with the pCDNA3-EGFP plasmid (Empty) or pCDNA3-EGFP-RhoA-T19N plasmid (RhoA DN) to inhibit RhoA-ROCK. An antibody to flotillin-1 was used to confirm the equal load of the membrane fraction. **E.** The total expression of E-cadherin or b-catenin was determined after treating MCF-7 cells with the indicated concentration of Y-27632 for 24 hrs through immunoblotting using the specific antibodies. **F.** Total expression level of E-cadherin was determined after transfecting cells with control si-RNA (scramble), si-RNA targeting ROCK1 (si-ROCK1) or ROCK2 (si–ROCK2) for 72 hrs through immunoblotting.

Epithelial cells bind together through the homophilic binding of E-cadherins, which form an intracellular molecular complex with several molecules, such as b-catenin and actin filaments [20], [21]. Thus, the loss of E-cadherin and b-catenin from the cell membrane was examined upon cell separation through ROCK inhibition using confocal microscopy. ROCK inhibition clearly resulted in the loss of the E-cadherin expression from the cell membrane [Fig. 3A]. The loss of E-cadherin and b-catenin, associated with ROCK inhibition from the cell membrane, were further confirmed through the constitutive overexpression of active RhoA (RhoA-CA) to activate ROCK or the dominant negative RhoA (RhoA-DN) to inhibit ROCK. MCF-7 cells overexpressing RhoA-DN induced the loss of E-cadherin and b-catenin from the cell membrane in immunolocalization assays [Fig. 3B]. However, MCF-7 cells overexpressing RhoA-CA presented strong E-cadherin and b-catenin expression at the cell membrane. In the Western blotting analysis, the E-cadherin membrane fraction was reduced after ROCK inhibition or the overexpression of RhoA-DN [Fig. 3C and D]. In addition, the total expression of E-cadherin and b-catenin was also down-regulated upon ROCK inhibition [Fig. 3E]. These results suggest that the disruption of cell junction upon ROCK inhibition might be associated with the loss of E-cadherin and b-catenin from the cell membrane.

### ROCK inhibition up-regulates Rac1 activity to disrupt cell junction

**Figure 4:**
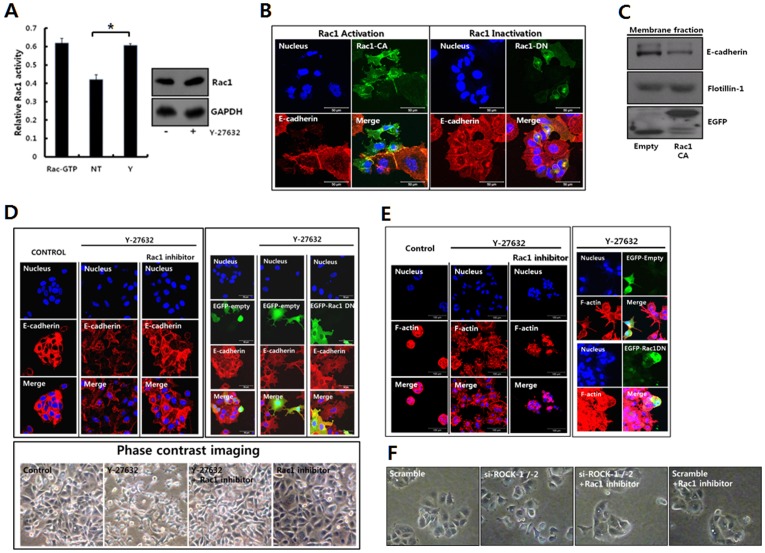
ROCK inhibition activates Rac1, which leads the disruption of the MCF-7 cell junction. **A.** MCF-7 cells were cultured on tissue culture plates for 48 hrs, and Y-27632 (20 µM) was added into the culture medium for 3 hrs. Equal amounts of cell lysates were used for the G-LISA Rac1 activity assay. The data are expressed as the means ± SD. n = 3 dishes. The p-value was less than 0.05 for comparisons between control (NT: no treatment) and treatment groups (Y: 20 µM Y-27632). *, *p*<0.05. Total Rac1 expression was determined through immunoblotting using a Rac1-specific antibody (right panel). **B.** MCF-7 Cells were transfected with plasmids for constitutively active Rac1 (EGFP-Rac1-Q61L, Rac1-CA) or dominant negative Rac1 (EGFP-Rac1-T17N, Rac1-DN). Subsequently, the fixed cells were analyzed through immunofluorescence staining using anti-E-cadherin antibody. The nuclei were stained with DAPI (blue). **C.** The E-cadherin expression in the membrane fraction was examined through immunoblotting using specific antibodies after transfecting MCF-7 cells with pCDNA3-EGFP plasmid or constitutively active Rac1 (EGFP-Rac1-Q61L, Rac1-CA). An antibody to flotillin-1 was used to confirm the equal load of the membrane fraction of the cell lysates. **D.** MCF-7 cells were seeded onto tissue culture plates. The next day, the cells were pretreated with Rac1 inhibitor (25 µM) for 1 hr, and Y-27632 (20 µM) was added to the indicated cell groups for 3 hrs (upper left panel). MCF-7 cells were transfected with EGFP plasmid or EGFP-Rac1-T17N plasmid (EGFP-Rac1 DN). Subsequently, Y-27632 (20 µM) was added into the indicated cell groups for 5 hrs (upper right panel). The cells were examined using phase contrast microscopy (lower panel) or confocal laser microscopy after immunofluorescence staining using anti-E-cadherin antibody (upper panel). **E.** MCF-7 cells were cultured in the Matrigel for 3D culture for 3 days. The cells were additionally cultured for 3 days with Y-27632 (20 µM) in the absence or presence of the Rac1 inhibitor (25 µM) (left panel). The cells were transfected with pCDNA3-EGFP plasmid (EGFP-Empty) or pCDNA3-EGFP-Rac1-T17N (EGFP-Rac1-DN) plasmid for 18 hrs prior to incubation in Matrigel. The cells were incubated in the Matrigel for 2 days. The cells were cultured for 2 additional days with Y-27632 (20 µM) or Rac1 inhibitor (25 µM) (right panel). The cells were fixed and analyzed through confocal laser microscopy after fluorescence staining using rhodamine-phalloidin. **F.** MCF-7 cells after transfection with control si-RNA (scramble) or si-RNAs targeting both ROCK-1 and -2 (si-ROCK1/2) were incubated for 48 hrs in the absence or presence of Rac1 inhibitor (20 µM). Cell morphology was observed using phase contrast microscopy.

We explored the molecular mechanism through which ROCK inhibition disrupts cell junction. We examined the involvement of Rac1, as it has been suggested that cell-to-cell junction through homophilic binding between E-cadherins is closely associated with Rac1 activity through an unknown molecular mechanism [22]–[27]. Previous reports demonstrating the down-regulation of the Rac1 activity through ROCK also substantiated the examination of Rac1 in this study for exploring the molecular mechanisms through which ROCK disrupts cell junction [28], [29]. As expected, Rac1 activity in MCF-7 cells was up-regulated upon ROCK inhibition although the expression of Rac1 was not altered upon ROCK inhibition [Fig. 4A]. The overexpression study confirmed that Rac1 activity is involved in the disintegration of the cell junction between MCF-7 cells. MCF-7 cells transfected with constitutively active Rac1 (Rac1-CA) showed the reduced expression of E-cadherin at cell membrane and detached cells from neighboring cells. However, the overexpression of dominant negative Rac1 (Rac1-DN) in MCF-7 cells did not change E-cadherin expression at the cell membrane and the disruption of cell junction [Fig. 4B]. The analysis of E-cadherin in the membrane fraction also clearly demonstrated the down-regulation of this protein through the overexpression of Rac1-CA [Fig. 4C]. To further elucidate the function of the increased Rac1 activity on ROCK inhibition in MCF-7 cells, Rac1 activity was inhibited using a specific inhibitor or through the transfection of Rac1-DN, concomitant with ROCK inhibition in 2D or 3D cultures [Fig. 4D and E]. Inhibiting Rac1 activity through treating cells with specific inhibitor or overexpressing Rac1-DN maintained E-cadherin expression at the cell membrane and decreasing the scattering of MCF-7 cells through ROCK inhibition on both 2D and 3D substrates, confirming that the disruption of cell junction through ROCK inhibition is ascribed to the increased Rac1 activity.

### ROCK inhibition of MCF-7 cells increases cell proliferation, migration, and invasion

**Figure 5:**
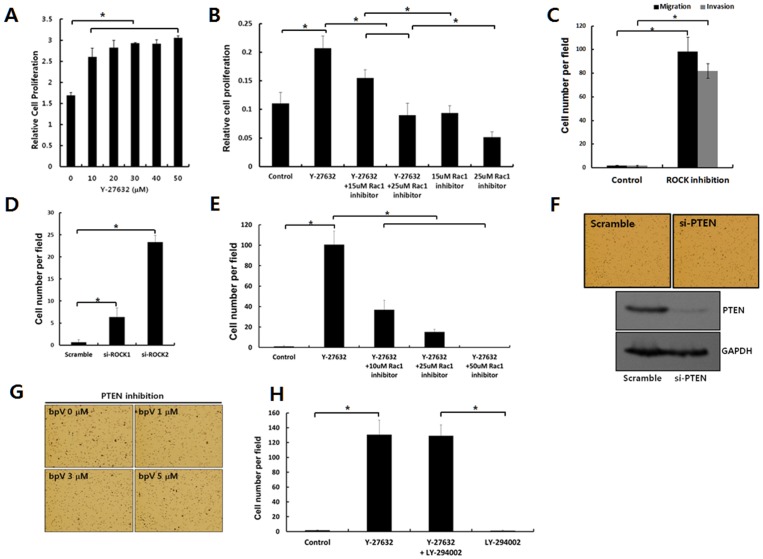
ROCK inhibition of MCF-7 cells increases cell migration and invasion through Rac1 activation. **A.** MCF-7 cells were cultured with the indicated concentration of Y-27632 for three days. The cell proliferation rate was subsequently measured through CCK-8 assay. The data are expressed as the mean ± SD. n = 3 dishes. The p-value was less than 0.05 for comparisons between un-treated and treatment groups (Y-27632) upon Krusal-Wallis test. *, *p*<0.05. **B.** MCF-7 cells were cultured with Y-27632 (20 µM) in the absence or presence of Rac1 inhibitor (15 or 25 µM) for three days. The cell proliferation rate was subsequently measured through CCK-8 assay. The data are expressed as the mean ± SD. n = 3 dishes. The p-value was less than 0.05 for comparisons between any two groups except comparison between the group of Y-27632 with 25 mM Rac1 inhibitor and the group of 15 mM Rac1 inhibitor upon one-way analysis of variance. *, *p*<0.05. **C.** The cells seeded on the Transwell chambers for the migration assay were incubated for 30 hrs in the absence or presence of Y-27632 (20 µM) to inhibit ROCK activity (upper panel). The cells seeded on the Transwell chambers covered with Matrigel for the invasion assay were cultured for 30 hrs in the absence or presence of Y-27632 (20 µM) to inhibit ROCK activity. The cells migrated into the lower chamber were manually counted after staining cells with hematoxylin. The data are expressed as the mean ± SD. n = 3 chambers. *, *P*<0.05 compared between groups treated with or without Y-27632. **D.** The cells transfected with control si-RNA (scramble), si-RNA targeting ROCK1 (si-ROCK1) or ROCK2 (si-ROCK2) were seeded on the Transwell chambers for migration assay and incubated for 48 hrs. The cells migrated onto the lower chamber were manually counted after staining cells with hematoxylin. The data are expressed as the mean ± SD. The p-value was less than 0.05 for comparisons between scramble group and transfected groups. *, *p*<0.05. **E.** The cells seeded on the Transwell chambers for migration assay were incubated with Y-27632 (20 µM) and the indicated concentration (10, 25 50 mM) of Rac1 inhibitor for 18 hrs. The cells migrated into the lower chamber were stained with hematoxylin. The cells migrated into the lower chamber were manually counted after staining cells with hematoxylin. The data are expressed as the mean ± SD. n = 3. The p-value was less than 0.05 for comparisons between Y-27632 group without Rac1 inhibition and any Y-27632 group with Rac1 inhibition. *, *p*<0.05. **F.** Cells transfected with control siRNA (scramble) or siRNA targeting PTEN (si-PTEN) were incubated for 2 days. Subsequently, the cells were replated into the Transwell chambers for migration assay. The cells migrated into the lower chamber for 24 hrs were stained with hematoxylin (upper panel). PTEN expression was analyzed through immunoblotting using specific antibodies against PTEN. **G.** The cells seeded in Transwell chambers for the migration assay were incubated with the indicated concentration of bpV (PTEN inhibitor; 1, 3, 5 µM) for 24 hrs. Cells migrated into the lower chamber were stained with hematoxylin. **H.** The cells were seeded in the Transwell chambers for the migration assay and incubated with ROCK inhibitors (Y-27632; 20 µM) and/or PI3-K inhibitors (LY-294002; 10 µM) for 24 hrs. The cells migrated into the lower chamber were manually counted after staining cells with hematoxylin. The data are expressed as the mean ± SD. n = 3. There was no statistical significance between ROCK inhibition groups (Y-27632) with / without PI3K inhibition (LY-294002).

For the activation of dormant cancer cells, cell proliferation, migration, and invasion should also be up-regulated concomitant with the disruption of cell junctions, which potentiates the dissipation of dormant cancer cells into surrounding tissues or contributes to the enlargement of the cancer mass. Cell proliferation was also accompanied by ROCK inhibition [Fig. 1B and Fig. 5A]. To examine the involvement of Rac1 up-regulation in the cell proliferation promoted by ROCK inhibition, the Rac1 activity of MCF-7 cells was inhibited by treating cells with a Rac1-specific inhibitor in concomittant with ROCK inhibition. Rac1 inhibition down-regulated the increased cell proliferation by ROCK inhibition in a dose-dependent manner [Fig. 5B]. These results indicate that Rac1 activation upon ROCK inhibition may also be involved in promoting the cell proliferation of MCF-7 cells.

As shown in the Fig. 2, disorganized and scattered cells were observed in the 3D Matrigel upon ROCK inhibition, suggested that ROCK inhibition up-regulates the migration and invasion of MCF-7 cells. Migration and invasion assays, using the Boyden chamber, clearly showed the up-regulation of cell migration and invasion upon ROCK inhibition [Fig. 1C, Fig. 5C and D]. Subsequently, we examined the molecules involved in the increased migration of MCF-7 cells upon ROCK inhibition. Rac1 is involved in the migration and invasion of epithelial cancer cells [30]. Thus, we determine whether the up-regulated Rac1 activity was associated with the increased migration / invasion of activated MCF-7 cells upon ROCK inhibition. The reversal of the increased migration upon ROCK inhibition through the dose-dependent treatment of MCF-7 cells with the Rac1 inhibitor [Fig. 5E], suggesting that Rac1 is associated with the increased migration of MCF-7 cells upon ROCK inhibition. These results further indicate that the dissociated cells might migrate and invade into the matrix to disseminate into the surrounding tissue upon ROCK inhibition. However, PTEN is not associated with the migration of MCF-7 cells. The inhibition of PTEN activity using a specific inhibitor or after transfecting cells with si-PTEN did not upregulate the cell migration [Fig. 5F and G]. The lack of PTEN involvement in cell migration was further confirmed by evidence that showed that the PI3-K inhibition did not down-regulate cell migration, which was increased by ROCK inhibition [Fig. 5H]. If PTEN was involved in promoting cell migration through the well-established up-regulation of the Akt phosphorylation [11], down-regulation of the Akt phosphorylation by PI3-K inhibition would decrease cell migration, which was up-regulated by PTEN inhibition.

### Typical EMT markers are not associated with MCF-7 cell activation

**Figure 6:**
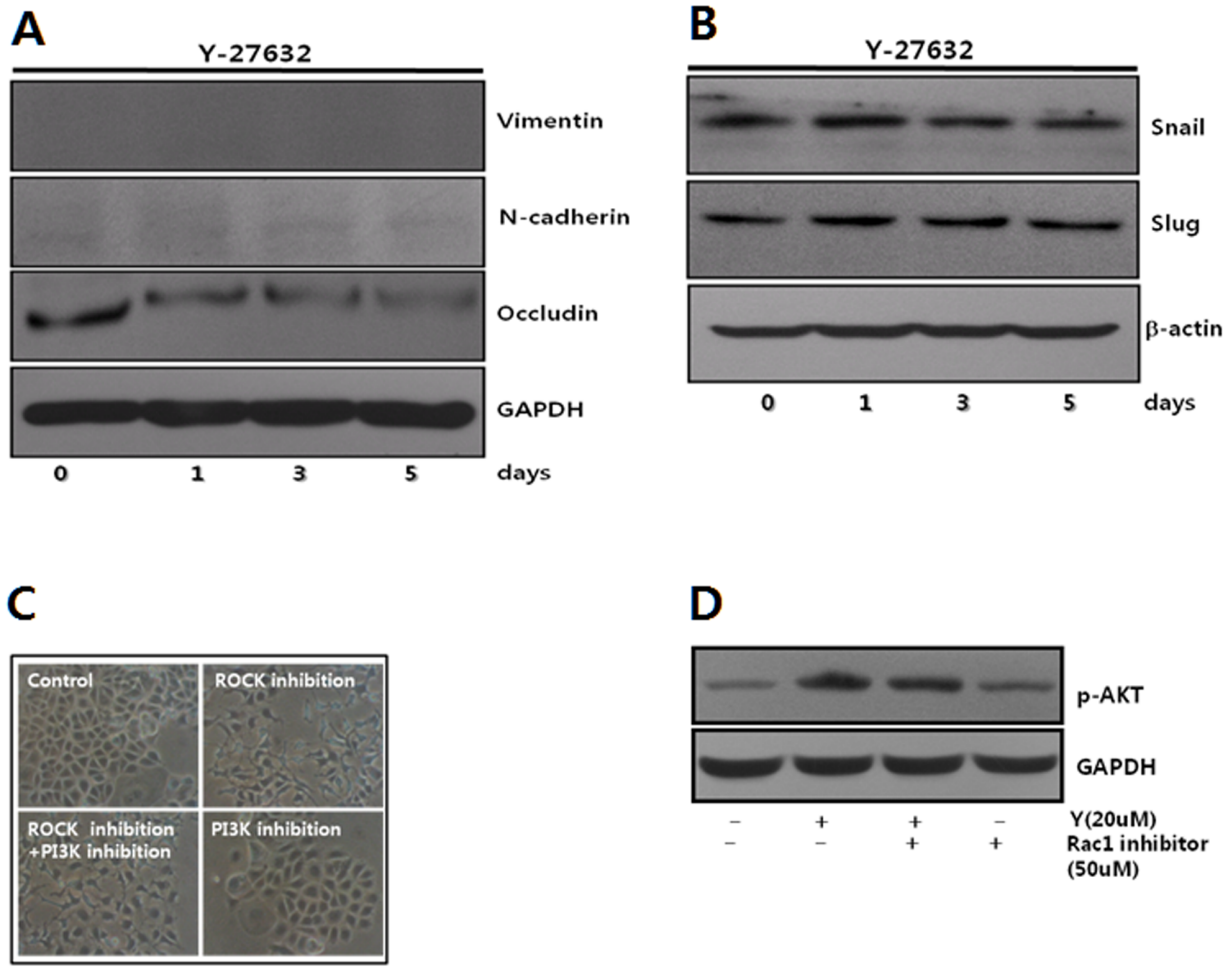
The activation of MCF-7 cells induced by ROCK inhibition follows non-typical EMT. **A and B.** Cells were cultured with Y-27632 (20 µM) for the indicated times. The cell lysates were immunoblotted to assess the expression levels of typical EMT marker proteins. **C.** The cells were treated with Y-27632 (20 µM) to ROCK activity and/or LY-294002 (10 µM) to inhibit PI3-K for 5 hrs. The morphology of the cells was observed through phase contrast microscopy. **D.** The cells were cultured for 24 hrs and treated with Y-27632 (20 µM) to inhibit ROCK in the presence or absence of Rac1 inhibitor (50 µM) for 3 hrs. Subsequently, the cell lysate from each group was immunoblotted to assess the phosphorylation levels of Akt using the anti-p-Akt antibody.

Alterations in EMT makers, such as vimentin, N-cadherin, slug, and snail, were examined upon ROCK inhibition, to determine whether cell scattering and increased cell migration / invasion upon ROCK inhibition occur through typical EMT. Western blotting did not show the up-regulation of these EMT markers, although the expression of occludin, a component of tight junctions [31], is reduced in cell cultures for 5 days [Fig. 6A and B]. These results indicate that the detachment of cells from neighboring cells upon ROCK inhibition does not occur through the typical EMT process.

Because the up-regulation of Akt induces EMT [32]–[34], we examined whether ROCK inhibition dissipated cells through the up-regulation of Akt. Although ROCK inhibition up-regulated Akt phosphorylation through PTEN inhibition [10], [11], cell dissipation induced through ROCK inhibition was not reversed after the down-regulation of Akt activity using the PI3-K inhibitor, LY-294002 [Fig. 6C]. These results indicate that Akt is not directly involved in cell dissipation upon ROCK inhibition. The up-regulated Akt activity upon ROCK inhibition, which was not down-regulated through Rac1 inhibition, also suggests that Akt activity is not associated with MCF-7 dissociation upon ROCK inhibition [Fig. 6D]. Taken together, these results indicate that the cell dissipation observed upon ROCK inhibition did not occur with the involvement of the PTEN pathway. In addition, the disintegration of MCF-7 cell junctions without the development of stress fibers upon ROCK inhibition also indicates that cell dissipation upon ROCK inhibition is not a typical EMT process [Fig. 2], although the development of stress fibers has been suggested as a marker of EMT [35].

### Lowered ROCK activity is associated with activation of MCF-7 cells on fibronectin substrate

**Figure 7:**
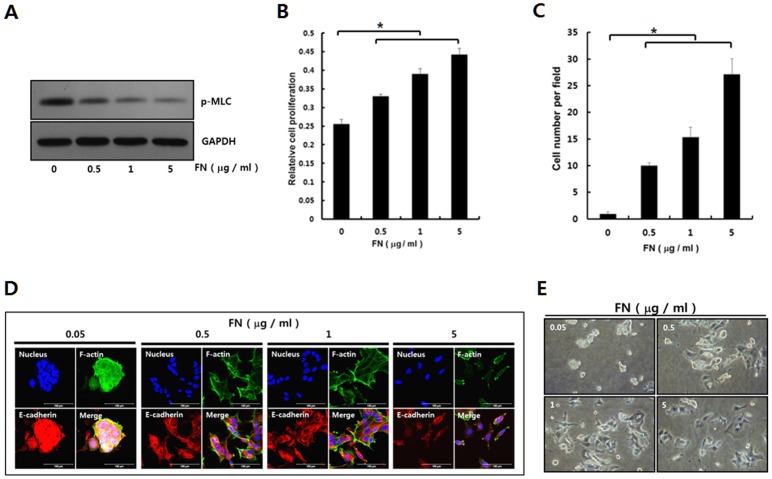
Adhesion strength has influence on ROCK activity and various cell activities in a dose dependent manner. **A.** To compare the relative levels of ROCK activation, depending on the amount of precoated FN, P-MLC expression levels were assessed through immunoblotting analysis using an anti-P-MLC antibody. **B.** The cells were cultured for three days on the hydrophobic culture dishes precoated with indicated amounts of fibronectin. Cell number was analyzed using CCK-8 kit. The data are expressed as the means ± SD. n = 3 dishes. The p-value was less than 0.05 for comparisons between any two groups. *, *p*<0.05. **C.** The cells seeded on the Transwell chambers precoated with the indicated amounts of fibronectin (FN) for the migration assay were incubated for 48 hrs. The cells migrated into the lower chamber were manually counted after staining cells with hematoxylin. The data are expressed as the mean ± SD. n = 3. The p-value was less than 0.05 for comparisons between any two groups. *, *p*<0.05. **D.** MCF-7 Cells were cultured for two days on hydrophobic culture dishes precoated with the indicated amounts of fibronectin (FN). The substrates were blocked with albumin (5% v/v) after coating with fibronectin. The localized patterns of F-actin and E-cadherin were examined using FITC-phalloidin and Cy3-E-cadherin through confocal laser microscopy. **E.** MCF-7 Cells were cultured for two days on hydrophobic culture dishes precoated with the indicated amounts of fibronectin (FN). The substrates were blocked with albumin (5% v/v) after coating with fibronectin. Cell morphology was examined using phase-contrast microscopy.

Next we explored the biological significance of the lowered ROCK activity in association with the activation of MCF-7 cells. In general, adhesion signaling is poor in dormant cancer cells [13], and weak adhesion signaling up-regulates RhoA-ROCK signaling [10], [11]. Thus, the ROCK activity in dormant MCF-7 cells was monitored on hydrophobic substrates precoated with various amounts of fibronectin to show a dependency on the strength of the adhesion signaling through an examination of the phosphorylated myosin light chain (p-MLC) [7]. Consistent with our hypothesis that ROCK activity was associated with the degree of adhesion signaling, the expression of p-MLC in MCF-7 cells, which reflects ROCK activity, decreased with increased adhesion signaling, and ROCK inhibition decreased the p-MLC expression in MCF-7 cells [Fig. 7A]. The MCF-7 cells presented the same phenotypes as shown for ROCK inhibition on the substrates precoated with fibronectin. Cell proliferation and migration were up-regulated on the substrates precoated with fibronectin in a dose dependent manner [Fig. 7B and C]. Furthermore, the E-cadherin expression at the cell-cell junction decreased on the fibronectin-coated substrates in a dose dependent manner [Fig. 7D], and the MCF-7 cells dissipated on the substrates precoated with a higher amount of fibronectin [Fig. 7D and E]. Based on these results, we can establish a hypothesis that the activation of MCF-7 cells may be associated with the ROCK activity down-regulated on the substrates with a higher adhesion strength. Further study is required to confirm this hypothesis.

## Discussion

This study demonstrates that ROCK inhibition induces MCF-7 dormant breast cancer cells to disseminate through the disintegration of cell junctions concomitant with increased cell proliferation, migration, and invasion when cultured on 2D or 3D substrates. The reduced expression of E-cadherin, b-catenin, and actin filament bundles at the cell membrane, which maintain cell junctions, might explain the molecular basis underlying the disassembly of MCF-7 cell junctions through ROCK inhibition. Thus, these results indicate a potential risk to activate dormant MCF-7 cells to metastasize to neighboring tissues or organs through ROCK inhibition. Thus, ROCK inhibition therapy should be cautiously administered, as there is a potential risk for the activation of dormant cancer cells. Furthermore, this study suggests that conditions, which decrease ROCK activity in MCF-7 cells, might result in the activation of dormant MCF-7 cells. Microenvironmental conditions that reduce ROCK activity might also be associated with conditions that up-regulate adhesion signaling [11]. Adhesion signaling in the microenvironment of cancer cells is up-regulated through the deposition of adhesion ligands or stiffening the matrix surrounding the cancer cells. Thus, the ectopic deposition of adhesion ligands or alterations in mechanical strength surrounding dormant cancer cells might be included as one of the causes re-activating dormant cancer cells. However, there are few reports showing the function of ROCK signaling in the cell junction, indicating that ROCK dissembles or loosens cell junctions. Thus, these reports suggest that ROCK inhibition might restrain tumor cell dissemination [36]–[38] rather than promote cell dissipation as shown in the present study. It is not clear in this moment why the results of the present study are different from those in previous reports showing the effect of ROCK on stabilizing the epithelial junction is not clear in this moment [36]–[39], excluding the cell type specificity or developmental stage in cancer cells; EMTs of the primary tumor cells *vs* dormant cancer cells. Dissembling cell junction through ROCK activation was observed in human ovarian cancer cells, colorectal adenocarcinoma cells, or normal human colonic epithelial cells. Interestingly, ROCK inhibition did not disrupt the cell junction of MCF-10A cells, a normal mammary gland epithelial line cell [Unpublished data]. Further study is needed to clarify the role of ROCK in maintaining cell junctions. In addition, the present study shows that the activation of cancer cells through ROCK inhibition is not evident in the mesenchymal type breast cancer cells, such as the MDA-MB-231 cells, which are more active than MCF-7 cells for the proliferation and migration rates. A dependency on responsiveness to the ROCK inhibition for the cell activity was previously reported in the normal mesenchymal cell type, such as osteoblasts [9]–[12]. ROCK inhibition had a small effect on the active MC3T3-E1 osteoblasts for the substrate with high adhesion strength and clearly stimulated the inactive cells for the substrate with low adhesion strength. These findings suggest that the responsiveness of cancer cells to ROCK inhibition may also vary and depend on the status of the cellular activity. Further study is required to clarify these differences in responsiveness to ROCK down-regulation which depends on the cell activity of cancer cells.

Notably, the un-altered expression of EMT markers, such as vimentin, slug, and snail, was observed despite the disruption of cell junctions and up-regulation of Akt phosphorylation after ROCK inhibition. The dissemination of MCF-7 cells upon ROCK inhibition on both 2D substrates and in the 3D matrix without the enhanced expression of EMT markers suggests that an untypical EMT might occur through ROCK inhibition. Thus, this study demonstrates that the up-regulated expression of snail or slug is not necessary to separate MCF-7 cells, and ROCK inhibition might be sufficient to dissociate MCF-7 cell junctions. The slight down-regulation of total E-cadherin upon ROCK inhibition, despite cell junction disruption, might reflect the unaltered expression of EMT markers, such as slug and snail, which regulate E-cadherin expression [40], [41]. Thus, an alternate mechanism for the metastasis of dormant cancer cells might exist, which does not follow the typical EMT process. Non-typical EMT might be included in the consideration of the routes of metastasis. The potential activation of dormant cancer cells should be evaluated when inhibiting ROCK signaling is considered for the treatment of various diseases, such as cardiovascular diseases [16], [17]. In addition, this finding substantiates further investigation into the down-regulation of ROCK activity, associated with the activation of dormant cancer cells. The solidified matrix might down-regulate ROCK activity in adjacent epithelial cells through up-regulating adhesion signaling.

The results of the present study demonstrated that Rac1 activation upon ROCK inhibition might be associated with disruption of MCF-7 cell junctions. Rac1 activation through ROCK inhibition in MCF-7 cells was expected, as ROCK antagonizes RhoA-dependent Rac1 activation in fibroblasts [42], and Rac inactivation through ROCK is occurs via FilGAP phosphorylation or ARHGAP22 activation in melanoma cells [43], [44]. Furthermore, ROCK inhibition increases Rac1 activity in osteoblast and astrocytoma cells [12], [38]. Accumulating evidence has shown that Rac1 plays a complex role in various cell activities, such as survival, hypertrophy, and remodeling [45], [46], in addition to the activities associated with actin-regulation, such as the formation of lamellipodia [47] and involvement in the migration and invasion of epithelial cancer cells [30]. The disruption of MCF-7 cell junctions upon ROCK inhibition is associated with up-regulated Rac1 activity, as these cells did not dissipate when the Rac1 activity was inhibited in the presence of a ROCK inhibitor. The disruption of cell junctions and morphological changes in MCF-7 cells from polyhedral to round or spindle shapes, as observed in the cells transfected with Rac1-CA and cells treated with Y-27632 to inhibit ROCK activity, further confirms the role of Rac1 in regulating cell junctions in MCF-7 cells. Y-27632 treatment might loosen E-cadherin, b-catenin, and cortical actin filament bundles from the cell membrane, reflecting the up-regulation of Rac1 upon ROCK inhibition, rather than the downregulating of total E-cadherin expression. The results of this study also suggest that the cell junctions of dormant MCF-7 breast cancer cells might be maintained in association with low Rac1 activity, which might be suppressed through potential high ROCK activity in dormant MCF-7 breast cancer cells. Further study is required to examine the level of RhoA activity in MCF-7 cells compared with normal epithelial cells. The results of the present study suggest the use of the Rac1 inhibitor to inhibit the activation of dormant cancer cells when the using of the ROCK inhibitor is inevitable for the clinical purposes. However, there are conflicting reports regarding the role of Rac1 in maintaining cell junctions and the invasion of cancer cells. Up-regulated Rac1 promotes the invasion of B-lymphoma cells and the metastasis of breast cancer cells [48], [49], whereas down-regulated Rac1 is associated with reduced E-cadherin expression and the reduced invasion of melanoma cells [50]. The cell-type specificity or developmental stage of cancer cells might explain the conflicting roles of Rac1 in cancer cells. Further study is needed to clarify the role of Rac1 in maintaining cell junctions.

MCF-7 cell dissipation upon ROCK inhibition might be further peculiar, as the upregulation of Akt activity is not directly involved in this process. The up-regulated Akt phosphorylation is associated with EMT. Constitutively active Akt induces EMT in squamous cell carcinoma cells [34]. The activation of Akt through leptin is associated with EMT in MCF-7 cells [51]. The expression of twist induces EMT through the activation of Akt in HELA and MCF-7 cells [52]. However, in the present study, the disintegration of cell junctions through ROCK inhibition is not directly associated with up-regulated Akt phosphorylation upon ROCK inhibition, as PI3-K inhibition did not reverse the effect of ROCK inhibition on the disruption of cell junctions. In addition, cell migration and invasion were also up-regulated through ROCK, which was previously shown in MCF-7 cells and strocytoma cells [53], [54]. The activation of cells in migration and invasion might substantially promote the dissipation of dissociated MCF-7 cells.

In summary, the results of the present study suggest that the cell junction of dormant MCF-7 breast cancer cells might be disrupted in association with the Rac1, which might be up-regulated upon ROCK inhibition. Increased Rac1 activity upon ROCK inhibition might also promote cell migration / invasion. Thus, activated MCF-7 cells upon ROCK inhibition might metastasize into the surrounding tissues or organs.
